# Proximal interruption of the pulmonary artery with systemic bronchial/intercostal aneurysm formation: a case report

**DOI:** 10.1186/s42155-025-00519-0

**Published:** 2025-08-01

**Authors:** Runlin Yang, Robert Ng, Albert Goh, Richard Pow

**Affiliations:** 1 Department of Interventional Radiology, Royal North Shore Hospital, Reserve Rd, St Leonards, NSW 2065 Australia; 2 Department of Interventional Radiology, Royal Prince Alfred Hospital, Camperdown, NSW 2050 Australia; 3 Sydney Medical School, University of Sydney, Camperdown, NSW 2050 Australia

**Keywords:** Proximal interruption of the pulmonary artery, Bronchial artery embolisation

## Abstract

Proximal Interruption of the Pulmonary Artery (PIPA) is a rare congenital condition with an incidence of 1 in 200,000–300,000 individuals. We report the case of a 67-year-old woman with PIPA who presented with massive haemoptysis. Imaging revealed a small calibre right main pulmonary artery, absence of upper/middle lobe pulmonary arteries, and tortuous right systemic collateral arteries. A multidisciplinary meeting favoured bronchial artery embolisation over right pneumonectomy, due to the bleeding risk associated with extensive transpleural systemic collateral arteries. The patient underwent two staged bronchial artery embolisation and remained free of haemoptysis at the most recent 13-month follow-up. This case highlights the potential for bronchial artery embolisation to serve as a first-line treatment in managing PIPA, as a less invasive alternative to surgery.

## Background

Proximal Interruption of the Pulmonary Artery (PIPA) is a rare congenital condition with an incidence rate ranging of 1 in 200,000 to 1 in 300,000 individuals [1]. Few case reports address both the radiological findings and management of PIPA. Here, we present a case of PIPA with massive haemoptysis, highlighting the challenging management.

## Case presentation

A 67-year-old female with a history of atrial fibrillation presented with large-volume haemoptysis (>200ml). She was on rivaroxaban, which was withheld on admission. The patient had been experiencing yearly episodes of haemoptysis since the age of 18, not previously investigated, though was aware of thoracic vascular abnormalities identified on a CT coronary angiogram in 2021.

**Fig. 1:**
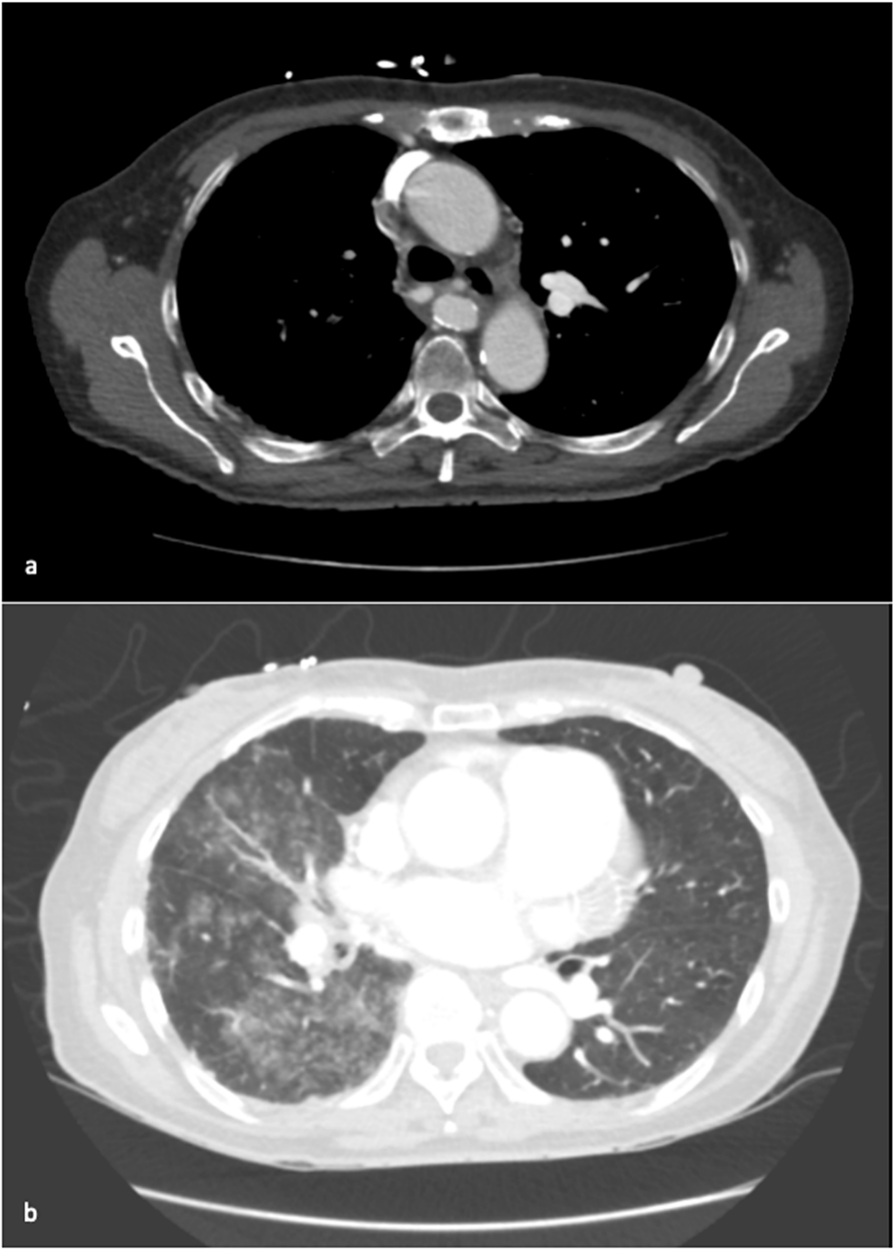
**a** CT bronchial-angiogram demonstrates aneurysm of the ICBT and hypertrophy of bronchial collaterals at the hilum; **b** CT bronchial-angiogram lung window demonstrates ground glass attenuation throughout right mid/lower lobes, consist with pulmonary haemorrhage at the time of presentation

**Fig. 2:**
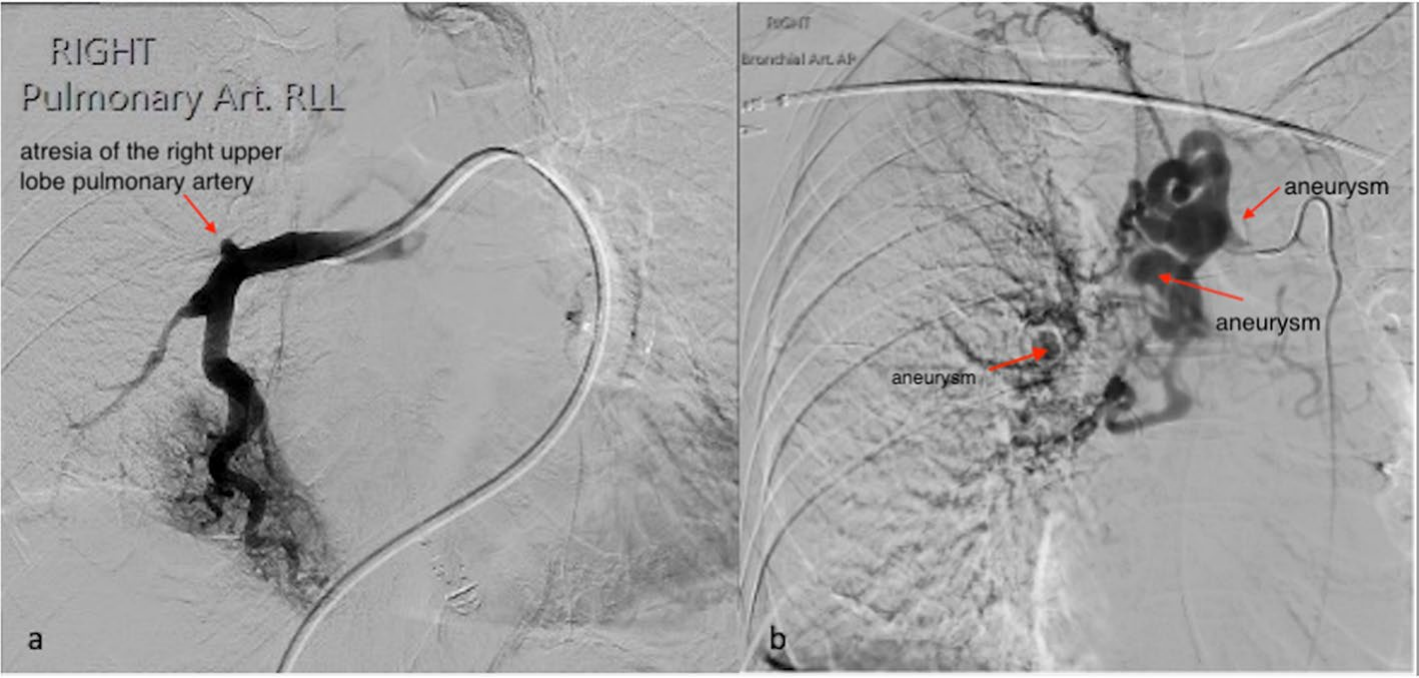
**a** Angiogram of right pulmonary artery demonstrated atresia of the right upper lobe pulmonary artery and a small right pulmonary artery supplying a portion of the right lower lobe; **b** Angiogram of the right ICBT demonstrates arterial hypertrophy and tortuosity with an aneurysm just beyond the origin measuring 19 × 20mm. Two further aneurysms are present including a sub-centimetre aneurysm just beyond the hilum

**Fig. 3:**
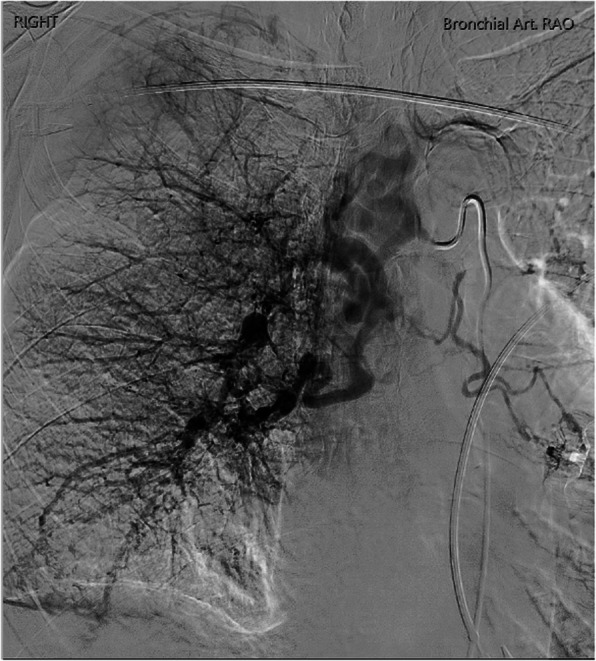
Delayed phase angiography demonstrated hypervascular parenchymal blush predominantly in the right upper to mid zones with drainage primarily via the right upper pulmonary vein

**Fig. 4:**
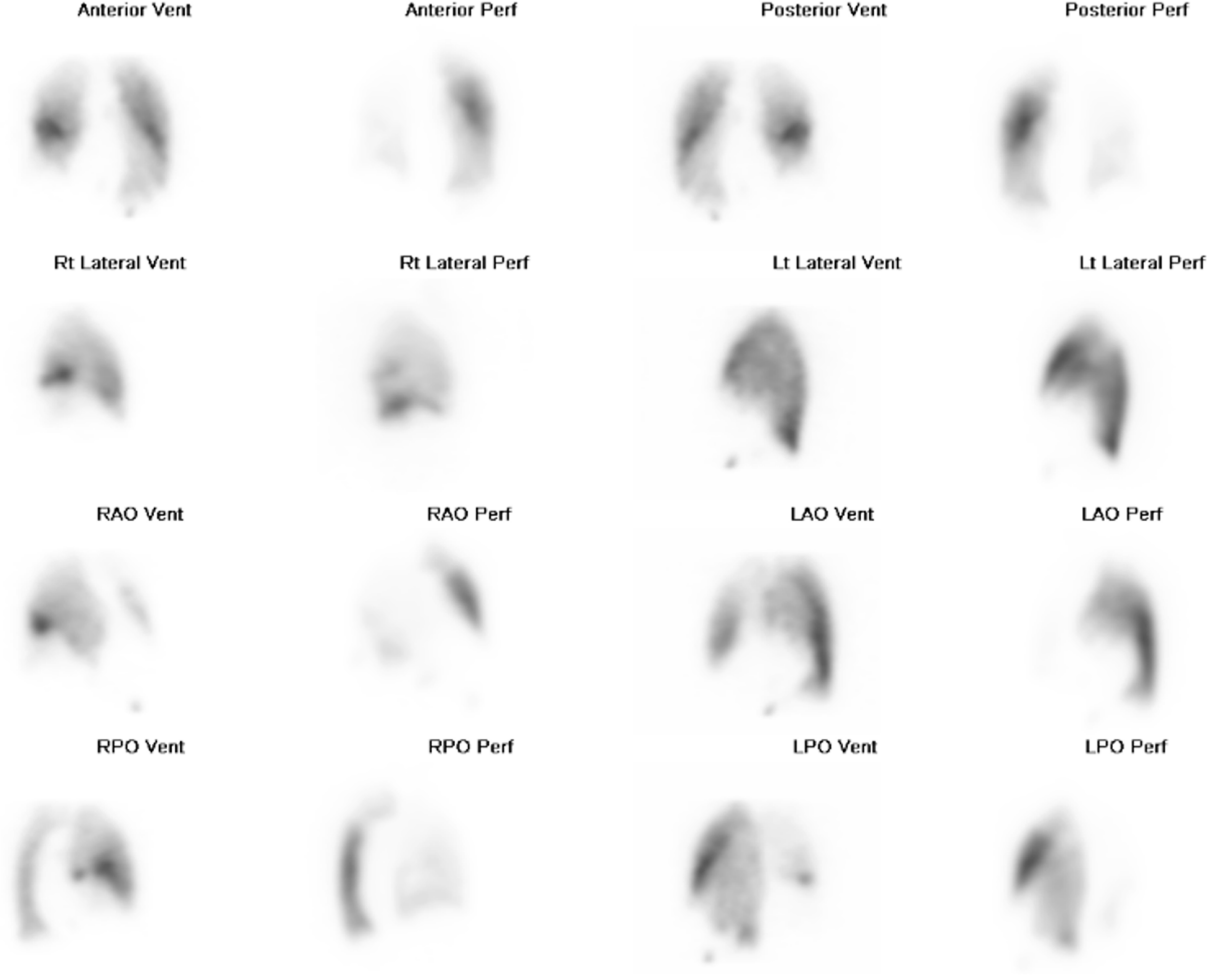
Ventilation-perfusion scan demonstrated limited ventilation to the right upper and middle lobe (16.6% and 7.1% respectively) with no perfusion (0%) to either. The right lower lobe received 23.6% ventilation and contributed to 5.7% of total perfusion

CT bronchial-angiogram demonstrated an aneurysm of the intercostal bronchial trunk (ICBT) and hypertrophy of bronchial collaterals at the hilum, and ground glass attenuation throughout right mid/lower lobes, consist with pulmonary haemorrhage at the time of presentation (Fig. 1). A subsequent diagnostic pulmonary arterial and systemic arterial angiogram demonstrated a normal left pulmonary artery and proximally interrupted small-calibre right main pulmonary artery with atresia of the right upper lobe artery and a small right pulmonary artery supplying only a portion of right lower lobe (Fig. 2a). The majority of the right lung received blood supply from extensive tortuous systemic collaterals, including the right ICBT, numerous right sided intercostal arteries, inferior phrenic artery, branches of the right internal mammary and thyrocervical trunk. There were numerous aneurysms present including a 19 x 20 mm aneurysm of the proximal ICBT and a further sub-centimetre aneurysm at the hilum (Fig. 2b). Delayed phase angiography demonstrated hypervascular parenchymal blush predominantly in the right upper to mid zones with drainage primarily via the right upper pulmonary vein (Fig. 3). Additionally, features of pulmonary arterial hypertension were observed including dilatation of the pulmonary trunk. A nuclear medicine ventilation-perfusion scan demonstrated limited ventilation to the right upper and middle lobe (16.6% and 7.1% respectively) with no perfusion (0%) to either. The right lower lobe received 23.6% ventilation and contributed to 5.7% of total perfusion (Fig. 4).

A multidisciplinary team meeting (MDT) decided that bronchial artery embolisation would be the preferred approach over surgical intervention, right pneumonectomy, due to the bleeding risk associated with extensive transpleural systemic collateral arteries. A staged embolisation approach was preferred first treating the ICBT including the aneurysmal segments followed by a second stage procedure targeting several other branches including dominant intercostal arteries.

**Fig. 5:**
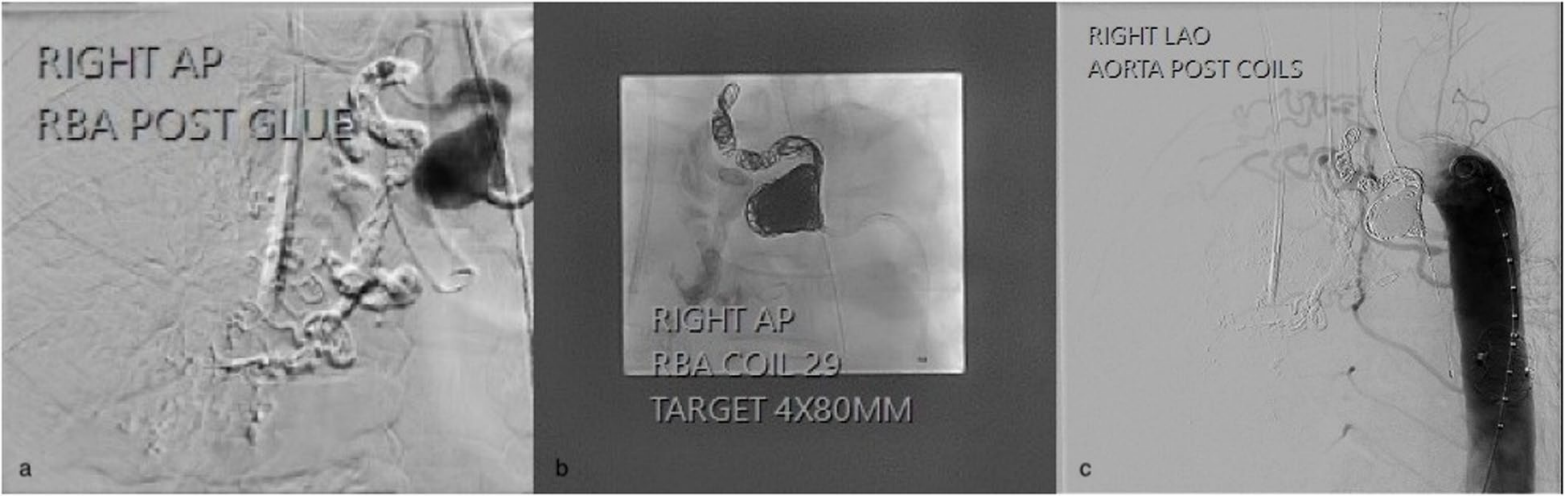
**a** Post ICBT embolisation used a mixture of Glubran2 (GEM) and lipiodol (1:4 ratio); **b** The proximal ICBT aneurysm was embolised with coils (Target, Stryker USA); **c** The final bronchial artery angiogram demonstrated successful occlusion of ICBT without any residual flow

The first bronchial artery embolisation was performed under general anaesthesia with double lumen intubation. Access to the ICBT was established via a transfemoral approach and triaxial technique (6 Fr Raabe sheath, 6 Fr AL2 guide catheter and 5 Fr Berenstein catheter), which confirmed the presence of a highly dilated ICBT with numerous aneurysms, most notably a large aneurysm near the ostium and at the hilum. No definite bronchial arterial to pulmonary vein fistula was observed. A microcatheter was advanced distally toward the hilar aneurysm and embolisation performed using a mixture of Glubran2 (GEM) and lipiodol (1:4 ratio), excluding the hilar aneurysm and the ectatic vessels more distally (Fig. 5a). The proximal ICBT aneurysm was then embolised with multiple coils (Target, Stryker USA) (Fig. 5b). The final angiographic assessment revealed successful occlusion of ICBT without any residual flow (Fig. 5c).

**Fig. 6:**
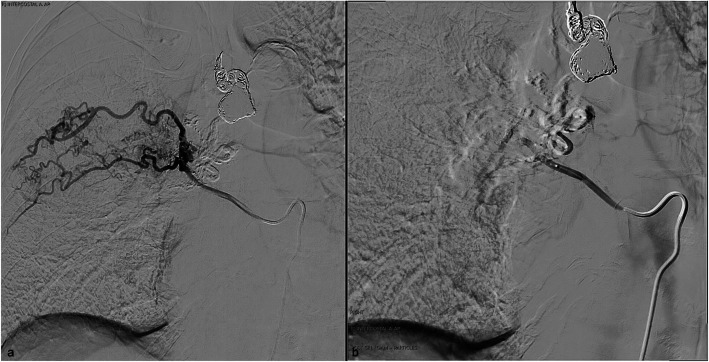
The second bronchial artery embolisation, targeting the two right posterior intercostal arteries with 500–700 μm polyvinyl alcohol (PVA) particles and gelatin sponge slurry. **a** Pre treatment; **b** Post treatment

During the patient’s recovery, an incidental left-sided pulmonary embolism was detected and treated with anticoagulation. She remained under close monitoring for 10 days, during which her hypertension was controlled, and no further episodes of haemoptysis occurred prior to discharge. Three months later, before the scheduled second-stage embolisation, the patient presented to the emergency department with recurrent haemoptysis. The subsequent second bronchial artery embolisation targeted the two right posterior intercostal arteries with 500–700μm polyvinyl alcohol (PVA) particles and absorbable gelatin sponge slurry (Fig. 6). The patient had an uneventful recovery, with no significant haemoptysis at the most recent 13-month follow-up.

PIPA is thought to arise from impaired development of the sixth aortic arch during embryogenesis [2] . A review of 108 patients with PIPA reported a median diagnosis age of 14 years, whilst our patient was diagnosed at the age of 67, making it a relatively unique case [2]. Right-sided PIPA is more common and typically occurs without associated congenital heart abnormalities, unlike left-sided PIPA, where 80% of patients have concurrent defects such as

Tetralogy of Fallot, atrial septal defect and ventricular septal defect [3]. The affected lung in PIPA receives blood supply from systemic collateral arteries, including the bronchial, intercostal, subclavian or internal mammary arteries [4].

Patients with PIPA may present with recurrent pulmonary infections, dyspnoea, chest pain and reduced exercise tolerance. Notably, 10-20% of these patients experience haemoptysis, primarily attributed to the rupture of thin-walled systemic pulmonary collateral arteries. In this case study, the patient experienced yearly episodes of mild haemoptysis before eventually presenting with a massive life-threating episode. Furthermore, pulmonary hypertension is observed in approximately 40% of PIPA patients [2].

Currently, there is no established consensus on the optimal treatment approach for PIPA, with most research focusing on radiological findings [5]. The treatment decisions for PIPA patients largely depend on their presenting symptoms. Kruzliaka et al’s review in 2013 discussed surgical interventions, such as revascularisation of the affected side, either through primary or staged pulmonary artery anastomosis, have been considered for paediatric patients [2]. However, in older patients revascularisation may not be feasible. Alternative surgical options include partial or total pneumonectomy. In PIPA patients, the physiological sequelae associated with hypertrophied aorto-pulmonary collaterals pose significant surgical risks, particularly of uncontrolled bleeding during pneumonectomy. This risk is generally higher in older individuals or patients with longstanding conditions, as their collateral arteries have had more time to develop and enlarge. A recent literature review by Mendogni et al. evaluated 26 adult patients with PIPA who underwent surgical pneumonectomy, with a mean age of 38 years [6]. Despite a zero mortality rate, 36% of patients experienced postoperative complications, with 12% of classified as Clavien–Dindo grade IIIb, defined as requiring additional intervention under general anaesthesia [6].In older individuals, such as our 67-year-old patient, it can be inferred that pneumonectomy may carry an even higher risk of morbidity.

Transarterial selective angioembolisation has been widely employed in patients with general haemoptysis, especially in recent years due to advancements in microcatheter and selective embolisation technique, showing favourable immediate technical and radiological success in 73-99% of cases [7]. In Mendogni et al’s literature review, the study reported that only 18% of patients who underwent transarterial embolisation experienced any postoperative complications, in contrast to 36% with pneumonectomy [6]. However, there remains a concern for recurrence of haemoptysis due to the extensive pulmonary collateral artery supply commonly seen in PIPA patients, resulting in a recurrence risk of up to 25% [8]. In case of recurrent haemoptysis, management typically involves repeat embolisation or surgery [9]. In addition, bronchial artery embolisation carries a rare but significant risk of inadvertent embolisation of a spinal artery, which can result in spinal cord ischemia and infarction. The incidence of spinal artery ischemia from bronchial artery embolisation has been reported to range from 1.4% to 6.5% [10]. To minimise this risk, using large particle PVA embolic agents greater than 350 μm is recommended [10]. In our case, we selected PVA particles sized 500–700 μm.

In our case study, an MDT discussion favoured transarterial embolisation due to its minimally invasive nature and risks associated with surgery. This case demonstrates that transcatheter embolisation is a safe and effective method for treating haemoptysis in this rare entity.

## Conclusion

This case highlights the challenges of managing PIPA with massive haemoptysis. Despite no consensus on the treatment of PIPA, our patient exhibited favourable outcomes following bronchial artery embolisation, which should be considered as potential first line treatment.

## Data Availability

The datasets used and/or analysed during the current study are available from the corresponding author on reasonable request

## Abbreviations

PIPA: proximal interruption of the pulmonary artery
ICBT: intercostal bronchial trunk
MDT: multidisciplinary team meeting
PVA: polyvinyl alcohol
